# Comparison of Magnetic Resonance Imaging–Based Risk Calculators to Predict Prostate Cancer Risk

**DOI:** 10.1001/jamanetworkopen.2024.1516

**Published:** 2024-03-07

**Authors:** Hiten D. Patel, Sebastiaan Remmers, Jeffrey L. Ellis, Eric V. Li, Monique J. Roobol, Andrew M. Fang, Petter Davik, Soroush Rais-Bahrami, Adam B. Murphy, Ashley E. Ross, Gopal N. Gupta

**Affiliations:** 1 Department of Urology, Feinberg School of Medicine, Northwestern University, Chicago, Illinois; 2 Department of Urology, Loyola University Medical Center, Maywood, Illinois; 3 Department of Urology, Erasmus MC Cancer Institute, University Medical Center Rotterdam, Rotterdam, the Netherlands; 4 Department of Urology, University of Alabama at Birmingham; 5 Department of Clinical and Molecular Medicine, Norwegian University of Science and Technology, Trondheim; 6 Department of Urology, St Olavs Hospital, Trondheim, Norway; 7 Department of Radiology, University of Alabama at Birmingham; 8 O’Neal Comprehensive Cancer Center, University of Alabama at Birmingham; 9 Department of Radiology, Loyola University Medical Center, Maywood, Illinois; 10 Department of Surgery, Loyola University Medical Center, Maywood, Illinois

## Abstract

**Importance:**

Magnetic resonance imaging (MRI)–based risk calculators can replace or augment traditional prostate cancer (PCa) risk prediction tools. However, few data are available comparing performance of different MRI-based risk calculators in external cohorts across different countries or screening paradigms.

**Objective:**

To externally validate and compare MRI-based PCa risk calculators (Prospective Loyola University Multiparametric MRI [PLUM], UCLA [University of California, Los Angeles]-Cornell, Van Leeuwen, and Rotterdam Prostate Cancer Risk Calculator–MRI [RPCRC-MRI]) in cohorts from Europe and North America.

**Design, Setting, and Participants:**

This multi-institutional, external validation diagnostic study of 3 unique cohorts was performed from January 1, 2015, to December 31, 2022. Two cohorts from Europe and North America used MRI before biopsy, while a third cohort used an advanced serum biomarker, the Prostate Health Index (PHI), before MRI or biopsy. Participants included adult men without a PCa diagnosis receiving MRI before prostate biopsy.

**Interventions:**

Prostate MRI followed by prostate biopsy.

**Main Outcomes and Measures:**

The primary outcome was diagnosis of clinically significant PCa (grade group ≥2). Receiver operating characteristics for area under the curve (AUC) estimates, calibration plots, and decision curve analysis were evaluated.

**Results:**

A total of 2181 patients across the 3 cohorts were included, with a median age of 65 (IQR, 58-70) years and a median prostate-specific antigen level of 5.92 (IQR, 4.32-8.94) ng/mL. All models had good diagnostic discrimination in the European cohort, with AUCs of 0.90 for the PLUM (95% CI, 0.86-0.93), UCLA-Cornell (95% CI, 0.86-0.93), Van Leeuwen (95% CI, 0.87-0.93), and RPCRC-MRI (95% CI, 0.86-0.93) models. All models had good discrimination in the North American cohort, with an AUC of 0.85 (95% CI, 0.80-0.89) for PLUM and AUCs of 0.83 for the UCLA-Cornell (95% CI, 0.80-0.88), Van Leeuwen (95% CI, 0.79-0.88), and RPCRC-MRI (95% CI, 0.78-0.87) models, with somewhat better calibration for the RPCRC-MRI and PLUM models. In the PHI cohort, all models were prone to underestimate clinically significant PCa risk, with best calibration and discrimination for the UCLA-Cornell (AUC, 0.83 [95% CI, 0.81-0.85]) model, followed by the PLUM model (AUC, 0.82 [95% CI, 0.80-0.84]). The Van Leeuwen model was poorly calibrated in all 3 cohorts. On decision curve analysis, all models provided similar net benefit in the European cohort, with higher benefit for the PLUM and RPCRC-MRI models at a threshold greater than 22% in the North American cohort. The UCLA-Cornell model demonstrated highest net benefit in the PHI cohort.

**Conclusions and Relevance:**

In this external validation study of patients receiving MRI and prostate biopsy, the results support the use of the PLUM or RPCRC-MRI models in MRI-based screening pathways regardless of European or North American setting. However, tools specific to screening pathways incorporating advanced biomarkers as reflex tests are needed due to underprediction.

## Key Points

**Question:**

How well do magnetic resonance imaging (MRI)-based risk calculators predict prostate cancer risk among adults in Europe and North America?

**Findings:**

In this diagnostic external validation study of 2181 patients from 3 unique cohorts, all 4 MRI-based risk calculators (Prospective Loyola University Multiparametric MRI [PLUM], UCLA [University of California, Los Angeles]-Cornell, Van Leeuwen, and Rotterdam Prostate Cancer Risk Calculator–MRI [RPCRC-MRI]) demonstrated good discrimination. The RPCRC-MRI and PLUM models had somewhat better calibration in the European and North American cohorts, while all models were prone to underestimate cancer risk in the advanced serum biomarker cohort.

**Meaning:**

The results support the use of RPCRC-MRI and PLUM in MRI-based screening pathways, but risk calculators incorporating advanced biomarkers are needed.

## Teaser

This diagnostic study compares discrimination and calibration for 4 magnetic resonance imaging (MRI)-based prostate cancer risk calculators within independent external cohorts from Europe and North America and a separate cohort with high utilization of an advanced serum biomarker.

## Introduction

Magnetic resonance imaging (MRI)–based risk calculators have emerged to replace or augment traditional prostate cancer (PCa) risk prediction models. The use of MRI changes PCa detection through both risk stratification as well as sampling of the prostate. The MRI-based PCa risk calculators have consistently outperformed traditional risk calculators, including the Prostate Biopsy Collaborative Group and European Randomized Study of Screening for Prostate Cancer 3 and 4 models.^1,2,3^ Two recent population-based randomized clinical trials, the STHLM3MR-2 (Prostate Cancer Detection Using the Stockholm3 Test and MR/​Fusion Biopsies)^4^ and GÖTEBORG-2 (GÖTEBORG Prostate Cancer Screening 2),^5^ have provided level 1 evidence on MRI to augment PCa screening, making the evaluation of MRI-based PCa risk calculators increasingly relevant.

While MRI-based models could be used to improve selection for prostate biopsy, numerous risk calculators exist, and few data are available to determine which models make the most accurate and consistent predictions across different populations and countries. First, some models lack external validation or have not made coefficients or equations publicly available, precluding the ability to make independent relative comparisons.^1^ Additionally, while African ancestry is incorporated as an established risk factor for PCa detection in many models developed in the US, European models generally do not account for potential differences by race and ethnicity, which may impact translatability.^6,7,8^ Last, PCa prevalence varies depending on the underlying risk factors in different populations, which may lead to inconsistent estimates. The exact prevalence among men evaluated for clinical suspicion of harboring PCa may not be known, or it may change over time with modifications to screening practices within a particular institution, city, or country. Specifically, the use of novel advanced biomarkers to augment prostate-specific antigen (PSA) screening may change the risk pool of patients selected to undergo MRI and/or biopsy, with patients at lower risk potentially forgoing one or both.^9,10,11,12,13^

Therefore, we compared diagnostic discrimination and calibration directly for the most promising MRI-based PCa risk calculators in the literature within independent external cohorts from Europe and North America. In addition, we compared the performance of the same models within a separate cohort with high utilization of an advanced serum biomarker, Prostate Health Index (PHI), as a reflex test in the PSA screening pathway.

## Methods

### Study Cohorts

This multi-institutional, retrospective diagnostic study was conducted among consecutive patients without a prior PCa diagnosis receiving multiparametric MRI before prostate biopsy. The European cohort consisted of patients from the Norwegian University of Science and Technology, Trondheim, Norway (January 1, 2016, to December 31, 2017); the North American cohort, patients from the University of Alabama at Birmingham Prospective MRI-Targeted Prostate Biopsy Cohort (January 1, 2015, to December 31, 2020); and the PHI cohort, patients from Northwestern Medicine hospitals affiliated with the Northwestern University Feinberg School of Medicine, Chicago, Illinois (January 1, 2018, to December 31, 2022). Institutional review board approval was obtained at each institution with a waiver of informed consent owing to the use of deidentified retrospective data. We followed the Transparent Reporting of a Multivariable Prediction Model for Individual Prognosis or Diagnosis (TRIPOD) reporting guideline.

The MRI scans were generally multiparametric and 3T in all cohorts except for very rare instances of 3T contraindications leading to 1.5T MRI in the North American cohort. Patients receiving prostate biopsy (transrectal or transperineal) after MRI were included in the present study. The MRIs were graded using the Prostate Imaging–Reporting and Data System (PI-RADS), version 2.0 (scores range from 1 to 5, with higher scores indicating greater suspicion for PCa).^14^ In the PHI cohort, PHI was used in more than 80% of patients during the decision to pursue MRI and/or biopsy, and biopsy was avoided in more than 30% of men who underwent MRI.^9^ Men who did not receive MRI and/or biopsy or who had a prior diagnosis of PCa were excluded for the present study. Complete case analysis was performed. Additional details specific to each cohort have been published previously.^3,9,10^ All cohorts were external to the models selected below.

### PCa Risk Calculator Model Selection

The 2 most promising MRI-based PCa risk calculator models from Europe and North America each were selected for evaluation based on publicly available model coefficients in the published literature or on request from authors, diagnostic discrimination (receiver operating characteristics to calculate area under the curve [AUC]) estimates in prior development and validation cohorts, and size of the sample used to develop the predictive models. For European models, the Van Leeuwen and Rotterdam Prostate Cancer Risk Calculator–MRI (RPCRC-MRI) were included based on consistent performance during external validation in prior studies.^6,7^ The RPCRC-MRI refers to the combined use of the MRI-augmented European Randomized Study of Screening for Prostate Cancer risk calculators 3 and 4.^7^ The MRI-based PCa risk calculators included from North America were the Prospective Loyola University Multiparametric MRI (PLUM) and UCLA (University of California, Los Angeles)–Cornell models.^3,11^ Other North American models were excluded based on prior evidence of lower performance on external validation or lack of agreement on sharing coefficients.^3,12,13^ Models were evaluated without recalibration, and predictions were calculated using published or author-provided coefficients. Validation cohorts represented actual clinical practice in each setting and differed in the degree of biopsy-naive patients and MRI characteristics compared with original development populations for models.

### Study Variables

Variables included were in line with requirements to obtain estimates from the PLUM, UCLA-Cornell, Van Leeuwen, and RPCRC-MRI models. Clinical variables included age, race and ethnicity, family history, digital rectal examination results, prior negative biopsy results, and PSA. The MRI variables included prostate volume and highest PI-RADS, version 2.0 lesion. Race and ethnicity were defined by self-report in the electronic medical record system and grouped as required by the models (African ancestry or no African ancestry). African ancestry has been associated with PCa risk and incorporated in some traditional (Prostate Cancer Prevention Trial Risk Calculator, version 2.0 and Prostate Biopsy Collaborative Group) and MRI-based risk calculators.

### Statistical Analysis

The primary outcome was diagnosis of grade group 2 or higher PCa, which was defined as clinically significant PCa. The AUCs and calibration plots were evaluated to compare the 4 selected models.

A decision curve analysis was conducted to estimate the clinical utility of each model based on net benefit across a broad predefined threshold range of 0 to 40% for harboring clinically significant PCa.^15^ All statistics and modeling were performed using R, version 4.2.1 (R Project for Statistical Computing) and Stata, version 15.0 (StataCorp LLC). Additional statistical details and code are provided in the eMethods in Supplement 1.

## Results

### Baseline Characteristics

A total of 303 patients were included in the European cohort (0 of African ancestry), 371 in the North American cohort (87 [23.5%] of African ancestry), and 1507 in the PHI cohort (189 [12.5%] of African ancestry), for an overall count of 2181 patients with a median age of 65 (IQR, 58-70) years and median PSA level of 5.92 (IQR, 4.32-8.94) ng/mL. The prevalence of any PCa and clinically significant PCa was 239 of 303 (78.9%) and 125 of 303 (41.3%), respectively, for the European cohort; 209 of 371 (56.3%) and 127 of 371 (34.2%), respectively, for the North American cohort; and 879 of 1507 (58.3%) and 718 of 1507 (47.6%), respectively, for the PHI cohort. Additional cohort details are given in Table 1, including biopsy-naive proportions of 239 patients (78.9%) for the European cohort, 178 (48.0%) for the North American cohort, and 1459 (96.8%) for the PHI cohort. Notably, the rate of biopsy-naive patients in the development cohorts for the PLUM model was 550 of 1010 (54.5%); for the UCLA-Cornell model, 1449 of 2354 (61.6%); and for the Van Leeuwen model, 344 of 393 (87.5%).^3,6,11^ While the development cohort for the RPCRC-MRI model had 504 of 961 (52.4%) biopsy-naive patients, models were developed separately for the biopsy-naive subgroup and the subgroup with a history of negative biopsy findings.

**Table 1.** Baseline Clinical Characteristics of the North American, European, and PHI Advanced Serum Biomarker Cohorts

Characteristic	Patient cohort			
	Overall (N = 2181)	North American (n = 371)	European (n = 303)	PHI (n = 1507)
Age, median (IQR), y	65 (50-70)	66 (61-71)	67 (62-70)	64 (57-69)
DRE results, No. (%)				
Abnormal	194 (8.9)	9 (2.4)	94 (31.0)	91 (6.0)
Normal	1946 (89.2)	362 (97.6)	168 (55.4)	1416 (94.0)
Unknown	41 (1.9)	0	41 (13.5)	0
PSA, median (IQR), ng/mL	5.92 (4.32-8.94)	6.66 (4.96-9.19)	8.50 (6.10-13.85)	5.36 (4.09-7.97)
MRI volume, median (IQR)	47 (34-67)	53 (37-78)	46 (33-66)	47 (34-65)
PSA density, median (IQR)	0.12 (0.08-0.20)	0.13 (0.08-0.19)	0.17 (0.11-0.33)	0.12 (0.08-0.20)
Biopsy history, No. (%)				
Biopsy naive	1876 (86.0)	178 (48.0)	239 (78.9)	1459 (96.8)
Prior negative	305 (14.0)	193 (52.0)	64 (21.1)	48 (3.2)
PI-RADS, No. (%)^a^				
1-2	286 (13.1)	15 (4.0)	107 (35.3)	164 (10.9)
3	479 (22.0)	113 (30.5)	39 (12.9)	327 (21.7)
4	925 (42.4)	139 (37.5)	54 (17.8)	732 (48.6)
5	491 (22.5)	104 (28.0)	103 (34.0)	284 (18.8)

Abbreviations: DRE, digital rectal examination; MRI, magnetic resonance imaging; PHI, Prostate Health Index; PI-RADS, Prostate Imaging–Reporting and Data System; PSA, prostate-specific antigen.

^a^ Higher scores indicate greater suspicion for prostate cancer.

### European and North American Cohorts

We found high diagnostic discrimination in the European cohort, with AUCs of 0.90 for the PLUM (95% CI, 0.86-0.93), UCLA-Cornell (95% CI, 0.86-0.93), Van Leeuwen (95% CI, 0.87-0.93), and RPCRC-MRI (95% CI, 0.86-0.93) models (Table 2). Notably, diagnostic discrimination for all 4 models was somewhat lower in the North American cohort, with AUCs of 0.85 (95% CI, 0.80-0.89) for the PLUM model and 0.83 for the UCLA-Cornell (95% CI, 0.80-0.88), Van Leeuwen (95% CI, 0.79-0.88), and RPCRC-MRI (95% CI, 0.78-0.87) models.

**Table 2.** AUC Estimates Based on Receiver Operating Curves for the 4 Selected Models in the North American, European, and PHI Advanced Serum Biomarker Cohorts

Model	Cohort, AUC (95% CI)		
	North American	European	PHI
PLUM	0.85 (0.80-0.89)	0.90 (0.86-0.93)	0.82 (0.80-0.84)
RPCRC-MRI	0.83 (0.78-0.87)	0.90 (0.86-0.93)	0.79 (0.77-0.81)
UCLA-Cornell	0.83 (0.80-0.88)	0.90 (0.86-0.93)	0.83 (0.81-0.85)
Van Leeuwen	0.83 (0.79-0.88)	0.90 (0.87-0.93)	0.80 (0.78-0.82)

Abbreviations: AUC, area under the curve; PHI, Prostate Health Index; PLUM, Prospective Loyola University Multiparametric Magnetic Resonance Imaging (MRI); RPCRC-MRI, Rotterdam Prostate Cancer Risk Calculator–MRI; UCLA, University of California, Los Angeles.

**Figure 1.**
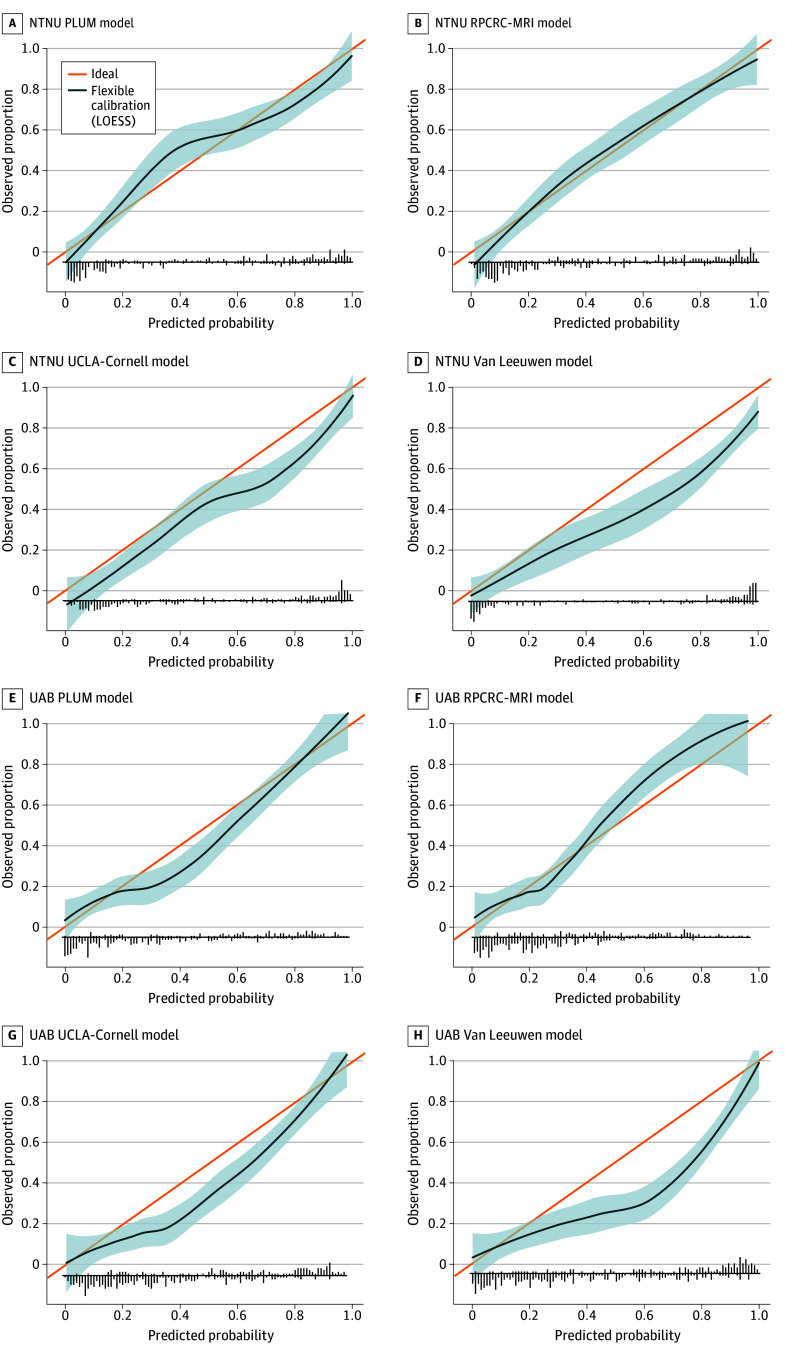
Calibration Plots for the Outcome of Clinically Significant Prostate Cancer in the European and North American Cohorts Cohorts are from the Norwegian University of Science and Technology (NTNU [A-D]) and the University of Alabama at Birmingham (UAB; North American cohort; E-H). The line for ideal represents perfect calibration, while the line for flexible calibration represents calibration of the model. Shaded areas represent 95% CIs, and vertical line segments plotted on the horizontal axis represent the frequency of cases with (above axis) and without (below axis) the event. LOESS indicates locally estimated scatterplot smoothing; PLUM, Prospective Loyola University Multiparametric Magnetic Resonance Imaging (MRI); RPCRC-MRI, Rotterdam Prostate Cancer Risk Calculator–MRI; and UCLA-Cornell, University of California, Los Angeles–Cornell.

The RPCRC-MRI model was best calibrated in the European cohort, followed by the PLUM model, which underpredicted risk within the predicted probability range of approximately 27% to 45% (Figure 1A-B). The UCLA-Cornell and Van Leeuwen models were prone to overprediction in the European cohort (Figure 1C-D). In the North American cohort, the PLUM and RPCRC-MRI models were reasonably calibrated with minor overprediction for PLUM (approximately 27% to 57% predicted probability range) and underprediction for RPCRC-MRI (approximately 50% to 80% predicted probability range) (Figure 1E-F). The UCLA-Cornell and Van Leeuwen models were prone to broader overprediction in the North American cohort (Figure 1G-H).

On decision curve analysis (eFigure in Supplement 1), all models provided similar net benefit in the European cohort, with lowest values across the 10% to 30% threshold for the UCLA-Cornell model. The net benefit for all models relative to a biopsy-all approach was somewhat lower in the North American cohort; there was a higher net benefit for the PLUM and RPCRC-MRI models at a threshold of greater than 22% compared with the UCLA-Cornell and Van Leeuwen models (similar benefit for all at a threshold probability of 10% to 22%).

### Advanced Serum Biomarker Screening Cohort

**Figure 2.**
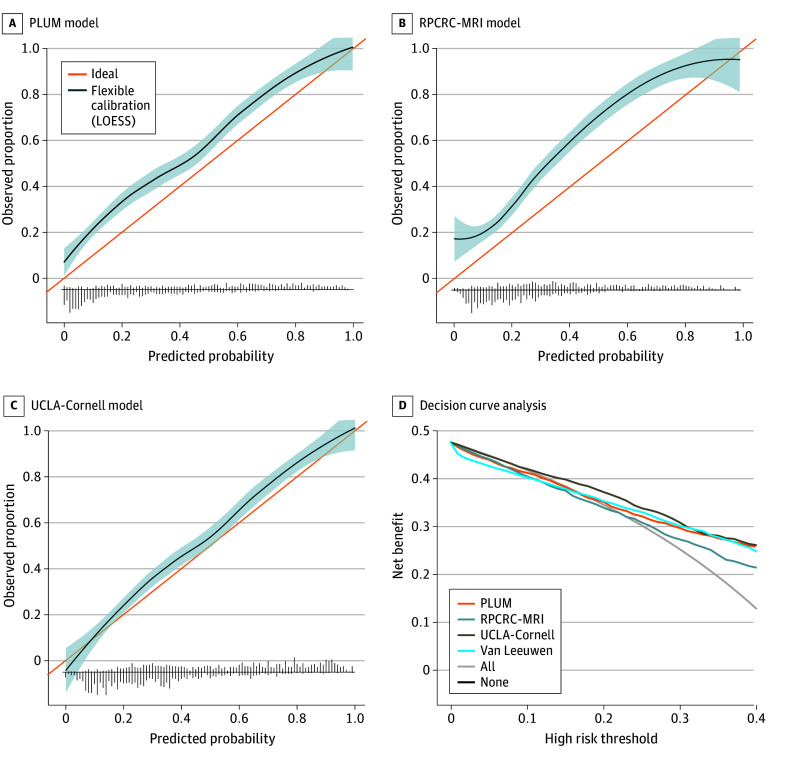
Calibration Plots for the Outcome of Clinically Significant Prostate Cancer and Decision Curve Analysis in the Prostate Health Index Advanced Serum Biomarker Cohort A through C, Calibration plots. The line for ideal represents perfect calibration, while the line for flexible calibration represents calibration of the model. Shaded areas represent 95% CIs, and vertical line segments plotted on the horizontal axis represent the frequency of cases with (above axis) and without (below axis) the event. The Van Leeuwen model was very poorly calibrated, with a negative slope and inability to calculate an intercept to obtain a calibration plot. D, Decision curve analysis. The high-risk threshold represents the threshold probability chosen where a biopsy would be advised. All represents an approach of biopsy for all patients, while none represents an approach of biopsy for no patients. Net benefit is the proportion of patients with true-positive results minus the proportion of patients with false-positive results weighted by the relative harm of false-positive and negative results. LOESS indicates locally estimated scatterplot smoothing; PLUM, Prospective Loyola University Multiparametric Magnetic Resonance Imaging (MRI); RPCRC-MRI, Rotterdam Prostate Cancer Risk Calculator–MRI; and UCLA-Cornell, University of California, Los Angeles–Cornell.

In the PHI cohort, AUCs for clinically significant PCa were slightly higher for the UCLA-Cornell model at 0.83 (95% CI, 0.81-0.85) and the PLUM model at 0.82 (95% CI, 0.80-0.84) compared with the European models with AUCs of 0.80 (95% CI, 0.78-0.82) for the Van Leeuwen and 0.79 (95% CI, 0.77-0.81) for the RPCRC-MRI models (Table 2). For calibration, all models were prone to underprediction, with the UCLA-Cornell model followed by the PLUM model exhibiting the best calibration (Figure 2A-C). Notably, the Van Leeuwen model was very poorly calibrated, with a negative slope and inability to calculate an intercept to obtain a calibration plot. On decision curve analysis, the UCLA-Cornell model demonstrated highest net benefit (Figure 2D). The PLUM and Van Leeuwen models exhibited similar net benefit from threshold probabilities of 15% to 40%, although Van Leeuwen was the lowest-performing model at threshold probabilities less than 15%.

## Discussion

In the last 5 years, data from numerous studies and clinical trials have supported the use of MRI prior to prostate biopsy, and this approach has been endorsed by both the European Association of Urology and American Urological Association for biopsy-naive patients and those with a history of negative biopsy findings.^16,17,18^ More recently, the STHLM3-MRI and GÖTEBORG-2 screening trials provided level 1 evidence on use of MRI for PCa screening, and while each study evaluated some unique parameters, a primary population of interest was based on a PSA level cutoff of 3 ng/mL or greater and a PI-RADS cutoff of 3 or greater.^4,5,19^ The MRI-based PCa risk calculators can provide more nuanced risk estimates for PCa screening by considering continuous values for PSA levels and PI-RADS rather than strict cutoffs and incorporating other “free” clinical risk factors such as age, family history, race and ethnicity, and prostate volume.

The present study externally validated and compared the performance of 4 promising MRI-based PCa risk calculators in independent cohorts from Europe and North America as well as a cohort that frequently used an advanced biomarker. Overall, we found all models performed well in the European and North American cohorts, with better calibration for the RPCRC-MRI and PLUM models over the UCLA-Cornell and Van Leeuwen models. However, the UCLA-Cornell and PLUM models performed slightly better than the others in the PHI cohort, with the UCLA-Cornell model demonstrating the best calibration.

Our results suggest that the RPCRC-MRI and PLUM models may be optimal choices for PCa risk prediction when MRI is used without additional advanced biomarkers to guide the decision to perform prostate biopsy. The differences in AUC or calibration between the RPCRC-MRI and PLUM models in the European or North American settings were minimal. However, all models in the present study were developed without the consideration of advanced biomarkers beyond PSA level in the screening paradigm.

In the PHI cohort, where PHI and MRI were both commonly considered before pursuing prostate biopsy, many patients at lower risk did not undergo biopsy. This likely contributed to the overall higher observed PCa risk relative to the predicted expected risk across all 4 models. The PHI cohort was also from North America, with the 2 North American models outperforming the European models in this setting, and the UCLA-Cornell model demonstrating the best calibration. The findings suggest the PHI cohort and UCLA-Cornell model development cohort may have come from settings with more similar screening practices relative to the other cohorts. Notably, while not a variable in the model, a subgroup of patients at UCLA did clinically use percentage of free PSA, which is 1 component of the PHI.

The implication of these findings is that while use of MRI-based PCa risk calculators is justified in conjunction with advanced biomarkers, screening could be further optimized if the advanced biomarker value was directly incorporated into the risk calculator.^20^ One study recently developed flexible models that consider additional data on percentage of free PSA levels or PHI, when available, in addition to MRI to make risk predictions for biopsy-naive men.^21^ The Stockholm3 test also serves as a proprietary risk calculator, including biomarker and clinical information without MRI, and although the risk calculator can be augmented with MRI data as an exercise, the additional value over sequential screening may be minor.^22^ Last, an alternative approach to screening would be to use a risk calculator solely among men with PI-RADS 3 lesions where the most uncertainty exists or consider forgoing systematic biopsy.^23,24^

A few prior studies^25,26,27^ have performed comparisons of MRI-based PCa risk models. Each of these studies has generally identified the European-based RPCRC-MRI or Van Leeuwen models as the most promising for clinical use, but the studies predated the publication of the North American PLUM and UCLA-Cornell models. Püllen et al^26^ included 307 European patients and found RPCRC-MRI provided the greatest net benefit among 3 assessed models, but the Van Leeuwen model was not evaluated. Lee et al^25^ included 449 Asian patients and found the Van Leeuwen model provided the greatest net benefit among 6 assessed models where RPCRC-MRI was also included. Finally, Saba et al^27^ included 468 European patients and found RPCRC-MRI followed by the Van Leeuwen models provided the greatest net benefit at a threshold probability of 15% among 4 assessed MRI-based models.

The present study includes a much larger overall sample of 2181 patients across European and North American cohorts while evaluating models from both settings. We verify prior results by demonstrating the RPCRC-MRI model performed well in a European cohort but extend the findings to the North American setting and show comparable performance to the PLUM model in both. Additionally, we found the Van Leeuwen model was poorly calibrated across all 3 settings we evaluated, arguing against its use. The results suggest an opportunity to consider recalibration of models prior to prospective clinical use if no well-calibrated model with preserved diagnostic discrimination exists in a given population. The PHI cohort is a unique setting where models have not been previously evaluated and constitutes a screening approach that may increase in practice.^20^ To our knowledge, the present study also provides the first external validation of the UCLA-Cornell risk calculator.^11^

### Limitations

This study has a few limitations that should be acknowledged. The study was retrospectively designed and conducted among patients receiving MRI and prostate biopsy. While general PCa screening practices in each cohort are known, the rate of PCa among men selected not to receive MRI and biopsy are unknown, with the evaluated models requiring complete data on both MRI and biopsy outcomes. Additional factors that could predict the presence of PCa, such as anterior lesion location, were not evaluated due to lack of inclusion in any evaluated model. Lastly, while a biopsy approach could affect PCa detection, prior studies have suggested that validation of models derived in the transrectal setting with patients who had received transperineal biopsy does not affect the performance of MRI-based PCa risk calculators.^2,25,26,27^ Despite the limitations, the study compared 4 promising MRI-based PCa risk prediction models to identify the optimal risk calculator choice in 3 distinct cohorts.

## Conclusions

In this diagnostic study, the PLUM, UCLA-Cornell, Van Leeuwen, and RPCRC-MRI risk calculators had good discrimination in the European (AUC, 0.90) and North American (AUCs, 0.83-0.85) cohorts, with better calibration for the RPCRC-MRI and PLUM models. In a cohort with high use of an advanced serum biomarker, all models were prone to underestimate clinically significant PCa risk, with highest AUC and calibration for the UCLA-Cornell model followed by the PLUM model. The results support the use of the PLUM or RPCRC-MRI models in MRI-based screening pathways regardless of a European or North American setting. However, tools specific to screening pathways incorporating advanced biomarkers as reflex tests are needed due to underprediction by available models.

## Notes

Supplement 1. **eMethods.** Study Cohorts and Statistical Methods and Codes **eFigure.** Decision Curve Analysis for the Outcome of Clinically Significant Prostate Cancer in Cohorts
eReferences

Supplement 2.
Data Sharing Statement
