# Supplementary material for: Comparison of Magnetic Resonance Imaging–Based Risk Calculators to Predict Prostate Cancer Risk
Source: JAMA Netw Open. 2024 Mar 7;7(3):e241516. doi: 10.1001/jamanetworkopen.2024.1516 (PMC10921249; doi:10.1001/jamanetworkopen.2024.1516)
Supplement: Supplement 1. — eMethods. Study Cohorts and Statistical Methods and Codes eFigure. Decision Curve Analysis for the Outcome of Clinically Significant Prostate Cancer in Cohorts eReferences [file jamanetwopen-e241516-s001.pdf]

Supplementary Online Content

Patel HD, Remmers S, Ellis JL, et al. Comparison of magnetic resonance imaging–based risk calculators to predict prostate cancer risk. *JAMA Netw Open*. 2024;7(3):e241516. doi:10.1001/jamanetworkopen.2024.1516

eMethods. Study Cohorts and Statistical Methods and Codes
eFigure. Decision Curve Analysis for the Outcome of Clinically Significant Prostate Cancer in Cohorts
eReferences

This supplementary material has been provided by the authors to give readers additional information about their work.

© 2024 Patel HD et al. *JAMA Network Open*.

eMethods. Study Cohorts and Statistical Methods and Codes

Study Cohorts

Patients included in each cohort were evaluated for clinical suspicion for PCa based on elevated PSA or DRE and had no prior diagnosis of PCa. Consecutive patients receiving prostate MRI followed by prostate biopsy were included during the timeframe specified for each cohort. There was no set PSA threshold or specific criteria defined in any cohort on when MRI would be obtained or biopsy performed as these were based on clinical practice of individual providers. Notably, all three cohorts included had implemented MRI-based screening paradigms during the time frame captured for the present study.

For the NA-Cohort and EU-Cohort, the majority of patients were expected to have received MRI prior to biopsy in each sample, but data on patients lacking MRI was not captured at these institutions. As an estimate from each institution, use of pre-biopsy MRI during the time frame captured in each cohort was about 60% for the NA-Cohort, 75% for the EU-Cohort. For the PHI-Cohort, the use of MRI prior to biopsy increased from 56% in 2018 to around 90% after 2020 as previously reported [1].

Statistical Methods

The predicted probability for presence of csPCa for each individual patient in the validation cohorts was calculated for each of the four included RCs. Variables in each cohort were categorized and arranged to match the original formatting required for each RC to allow computation. Coefficients of the risk models were used to calculate the linear predictors of each specific model. For multinomial models such as PLUM, two values were summed (h1 and h2) and exponentiated to obtain the estimated probability of csPCa ((EXP(h2))/(1+EXP(h1) + EXP(h2)). In the code below, h1 is referred to as "df1$lp_plum_indolent" while h2 is referred to as "df1$lp_plum_cspca". Receiver operating characteristics were used to assess discrimination by calculating area under the curve (AUC). Discrimination represents the degree to which the model can distinguish patients with and without csPCa.

Calibration was evaluated by comparing observed outcomes to predicted probabilities [2]. Figures 1 and 2 include calibration plots showing ascending predicted probabilities for csPCa against actual observed frequencies. A non-linear, flexible plot with 95% confidence bands was employed.

The decision curve analysis in eFigure 1 and Figure 2C evaluated the net benefits of models against a default approach of prostate biopsy for all of the included saple and flat horizontal line of zero net benefit for prostate biopsy for none. A threshold probability is plotted on the x-axis to represent what risk threshold may be sufficient to consider prostate biopsy. Prior studies have considered values between 10% to 30% [3]. The net benefit is the proportion of patients who are true positives minus the proportion of patients who are false positives with the latter weighted by the relative harm of false positive and negative results (determined by the chosen threshold probability) [4].

Statistical Code

<u>General description of STATA version 15.0 (STATA Corp, College Station, TX) code used in Table 1 tabulations:</u>

```
sum age, d

tab dre

sum psa, d

sum mri_p_volume, d

sum psad, d

tab prior_neg_n

tab piradcat
```

General description of R version 4.2.1 code used for analysis of discrimination and calibration (using PLUM as the example):

```
rm (list = ls())

setwd("V:/Users/039757Remmers/Bestanden_urologie/ERSPC/RC validations/20210804_Patel")

library(CalibrationCurves)

library(rmda)

df <- "" #American I cohort

df1 <- "" #European cohort

df2 <- "" #American II cohort

## PLUM

df1$MRI_PLUM_GG1 <- ifelse (df1$PIRADS_score <= 2, 0,
                        ifelse (df1$PIRADS_score == 3, 0.353384,
                              ifelse (df1$PIRADS_score == 4, 0.949164,
                                    ifelse (df1$PIRADS_score == 5, 1.874741, NA))))

df1$MRI_PLUM_GG2 <- ifelse (df1$PIRADS_score <= 2, 0,
                        ifelse (df1$PIRADS_score == 3, 0.4550155,
                              ifelse (df1$PIRADS_score == 4, 2.210478,
                                    ifelse (df1$PIRADS_score == 5, 3.634756, NA))))

df1$lp_plum_indolent <- with(df1, -2.397652 + MRI_PLUM_GG1 + age * .0350792 + 0 + 0 + prev_bx * -1.17898 + log(PSAD) * .1668917 + `Prostate volume by MRI (ml)`*-0.0186987)

df1$lp_plum_cspca <- with(df1,-3.492468 + MRI_PLUM_GG2 + age * 0.0772189 + 0 + 0 + prev_bx *-1.15653 + log(PSAD) * 1.204331 + `Prostate volume by MRI (ml)`*-0.0201334)
```

© 2024 Patel HD et al. *JAMA Network Open*.

```
df1$prob_indolent <- with(df1, exp(lp_plum_indolent) / (1+ exp(lp_plum_cspca) + exp(lp_plum_indolent)))

df1$prob_cspca <- with(df1, exp(lp_plum_cspca) / (1+ exp(lp_plum_cspca) + exp(lp_plum_indolent)))

df1$prob_no <- with(df1, 1 / (1+ exp(lp_plum_cspca) + exp(lp_plum_indolent)))

df1$prob_any <- df1$prob_indolent + df1$prob_cspca

df$cohort <- "UAB"

df1$cohort <- "NTNU"

df2$cohort <- "NW"

names(df2) <- names(df1) <- names(df)

df <- do.call(rbind, list(df, df1, df2))

val.prob.ci.2(p = df$plumcspca[df$cohort == "UAB"], y = df$cspca[df$cohort == "UAB"], statloc = F, dostats = F, main = "PLUM\ncsPCa")

val.prob.ci.2(p = df$plumcspca[df$cohort == "NTNU"], y = df$cspca[df$cohort == "NTNU"], statloc = F, dostats = F, main = "PLUM\ncsPCa")

val.prob.ci.2(p = df$plumcspca[df$cohort == "NW"], y = df$cspca[df$cohort == "NW"], statloc = F, dostats = F, main = "PLUM\ncsPCa")

cols <- RColorBrewer:: brewer.pal(3, "Set1")

cols <- cols[-1]

dca.model3 <- decision_curve(cspca ~ plumcspca, data = df[df$cohort == "UAB", ],

                             fitted.risk = T,

                             study.design = "cohort")

dca.model3 <- decision_curve(cspca ~ plumcspca, data = df[df$cohort == "NW", ],

                             fitted.risk = T,

                             study.design = "cohort")

dca.model3 <- decision_curve(cspca ~ plumcspca, data = df[df$cohort == "NTNU", ],

                             fitted.risk = T,

                             study.design = "cohort")

plot_decision_curve(list(dca.model3),

                    curve.names = "PLUM",

                    cost.benefit.axis = F,

                    col = cols[1],
```

© 2024 Patel HD et al. *JAMA Network Open*.

```
            lty = 1,
            confidence.intervals = F,
            standardize = F,
            xlim = c(0, .4))
title("NTNU\nPrediction csPCa")
library(pROC)
as.numeric(auc(cspca ~ plumcspca, data = df[df$cohort == "UAB", ]))
ci.auc(auc(cspca ~ plumcspca, data = df[df$cohort == "UAB", ]), method = "bootstrap")
as.numeric(auc(cspca ~ plumcspca, data = df[df$cohort == "NTNU", ]))
ci.auc(auc(cspca ~ plumcspca, data = df[df$cohort == "NTNU", ]), method = "bootstrap")
as.numeric(auc(cspca ~ plumcspca, data = df[df$cohort == "NW", ]))
ci.auc(auc(cspca ~ plumcspca, data = df[df$cohort == "NW", ]), method = "bootstrap")
```

© 2024 Patel HD et al. *JAMA Network Open*.

eFigure. Decision Curve Analysis for the Outcome of Clinically Significant Prostate Cancer in Cohorts

Cohorts are from the A) Norwegian University of Science and Technology (NTNU; EU-Cohort) and B) University of Alabama (UAB; NA-Cohort). The "High Risk Threshold" represents the threshold probability chosen where a biopsy would be advised. "All" represents an approach of biopsy for all patients while "None" represents an approach of biopsy for no patients. "Net Benefit" is the proportion of patients who are true positives minus the proportion of patients who are false positives weighted by the relative harm of false positive and negative results.

A)

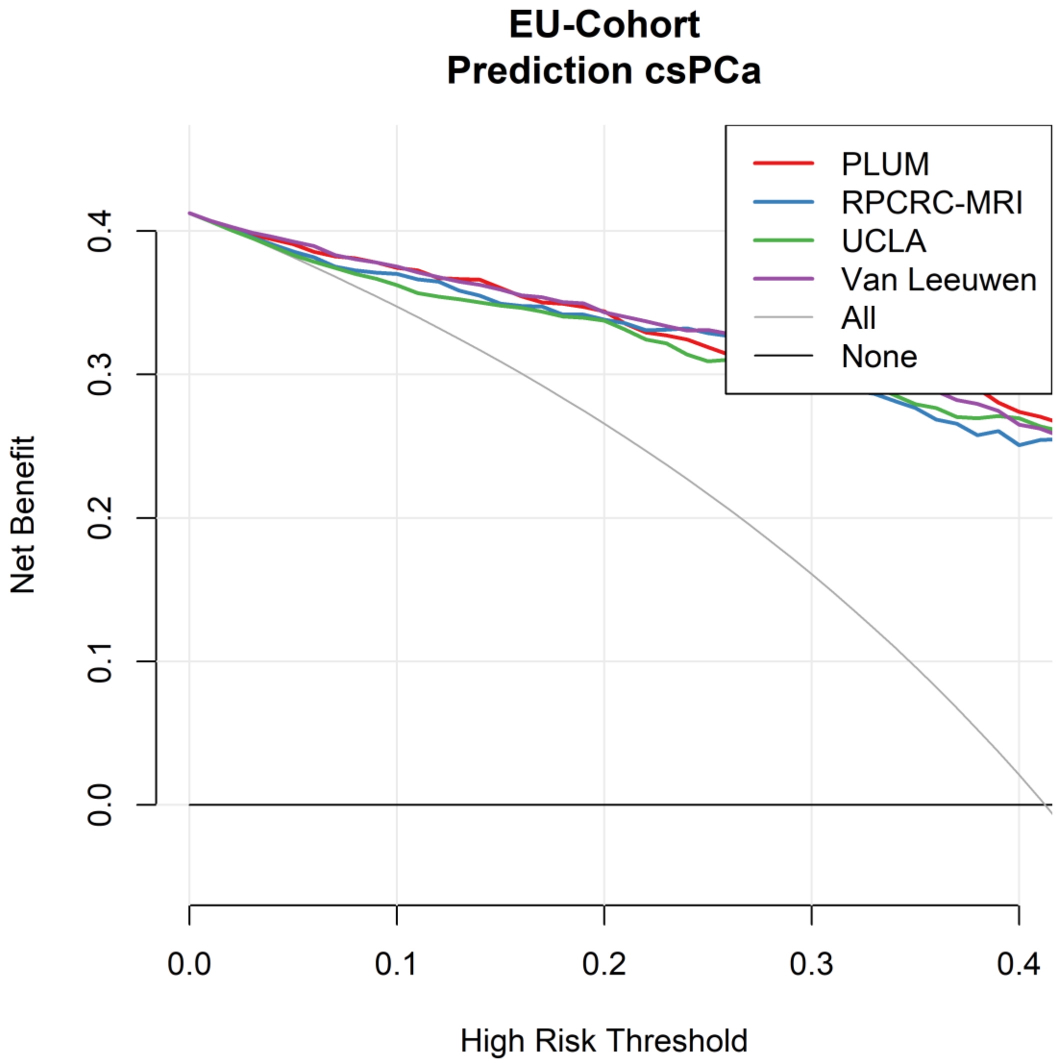

© 2024 Patel HD et al. *JAMA Network Open*.

B)

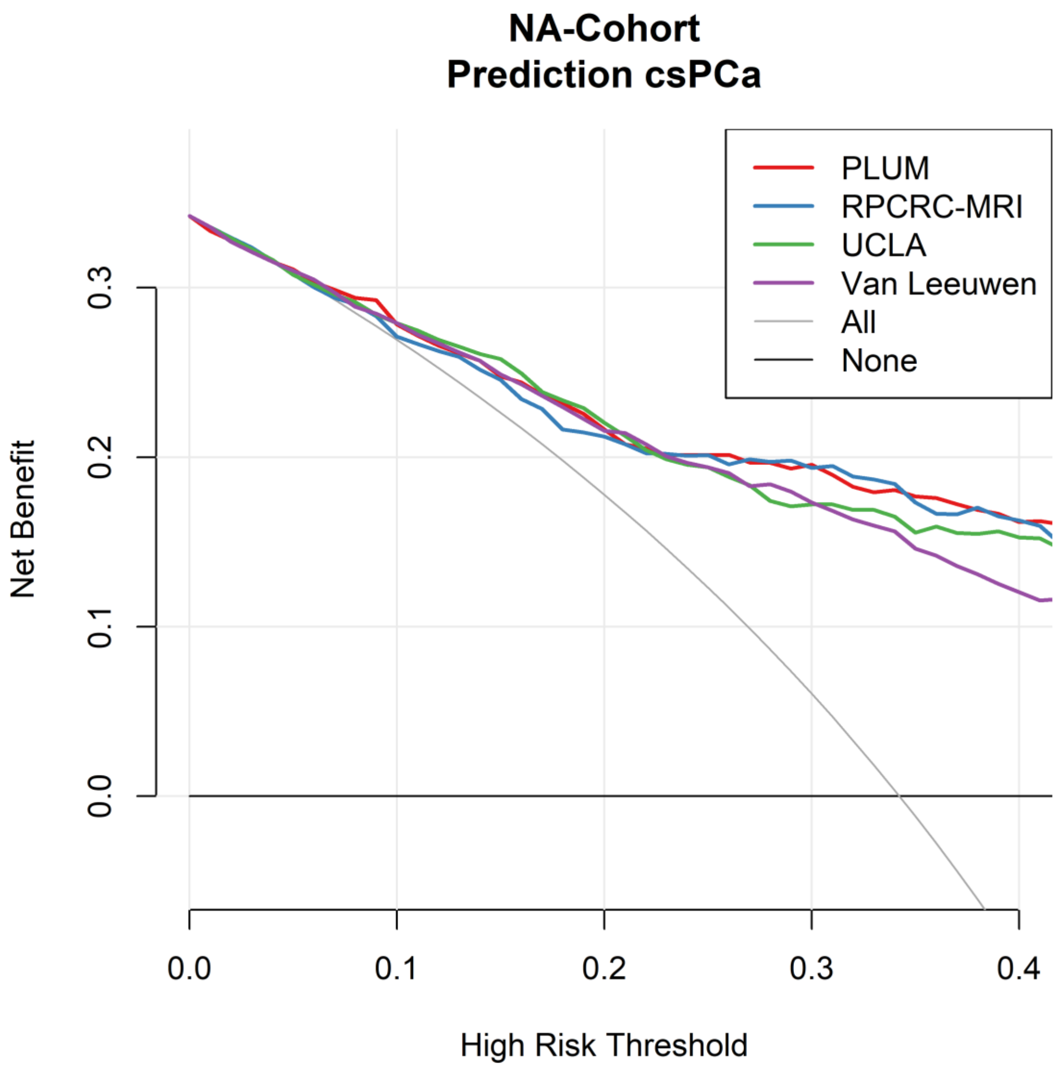

© 2024 Patel HD et al. *JAMA Network Open*.

eReferences

1. Siddiqui MR, Ansbro B, Shah PV, et al. Real-world use of MRI for risk stratification prior to prostate biopsy. Prostate Cancer Prostatic Dis. 2023 Jun;26(2):353-359.
2. Van Calster B, McLernon DJ, van Smeden M, et al. Calibration: the Achilles heel of predictive analytics. BMC Med. 2019 Dec 16;17(1):230.
3. Patel HD, Koehne EL, Shea SM, et al. A prostate biopsy risk calculator based on MRI: development and comparison of the Prospective Loyola University multiparametric MRI (PLUM) and Prostate Biopsy Collaborative Group (PBCG) risk calculators. BJU Int. 2023 Feb;131(2):227-235.
4. Vickers AJ, Elkin EB. Decision curve analysis: a novel method for evaluating prediction models. Med Decis Making. 2006;26(6):565-574.

© 2024 Patel HD et al. *JAMA Network Open*.
