# Supplementary material for: Comparison of Magnetic Resonance Imaging–Based Risk Calculators to Predict Prostate Cancer Risk
Source: JAMA Netw Open. 2024 Mar 7;7(3):e241516. doi: 10.1001/jamanetworkopen.2024.1516 (PMC10921249; doi:10.1001/jamanetworkopen.2024.1516)
Supplement: Supplement 2. — Data Sharing Statement [file jamanetwopen-e241516-s002.pdf]

Data Sharing Statement

Patel. Comparison of Magnetic Resonance Imaging–Based Risk Calculators to Predict Prostate Cancer Risk. *JAMA Netw Open*. Published March 07, 2024.
doi:10.1001/jamanetworkopen.2024.1516

Data
Data available: Yes
Data types: Deidentified participant data
How to access data: hiten.patel@nm.org
When available: With publication

Supporting Documents
Document types: None

Additional Information
Who can access the data: Researchers whose proposed use of the data has been approved
Types of analyses: For approved analytic plans
Mechanisms of data availability: After approval of a proposal
Any additional restrictions: Data use dependent on approval by contacts at each institution
